# Gay Dating App Users Support and Utilize Sexual Health Features on Apps

**DOI:** 10.1007/s10461-021-03554-9

**Published:** 2022-01-11

**Authors:** Jennifer Hecht, Maria Zlotorzynska, Travis H. Sanchez, Dan Wohlfeiler

**Affiliations:** 1 Building Healthy Online Communities, Springboard HealthLab, 5601 Van Fleet Ave, Richmond, CA 94804 USA; 2 Rollins School of Public Health, Emory University, 1518 Clifton Road, Atlanta, GA 30322 USA

**Keywords:** MSM, HIV, Sexual health, Internet, Dating apps

## Abstract

Men who have sex with men (MSM) frequently meet sex partners through dating apps. Research has demonstrated an association between app use and greater number of sex partners and STIs, but dating apps also pose an opportunity for intervention. By advocating for sexual health features on dating apps, Building Healthy Online Communities (BHOC) aims to increase communication about sexual health among app users. In partnership with Emory University, BHOC added questions to an annual survey of MSM. The questions assessed awareness and uptake of profile fields and sexual health features on the dating apps. Among survey participants, 67% (6737/10,129) reported using dating apps to meet a partner in the past year. Among this group, 77% (4993/6525) reported awareness of sexual health features. 61% of app users (2866/4721) who were aware of them reported using one or more sexual health features. BHOC continues to advocate for increased uptake of these features.

## Introduction

Men who have sex with men (MSM) continue to experience a disproportionate burden of HIV infection [1, 2]. MSM were early adopters of using the internet to meet sexual and romantic partners, going back to the early 2000s [3, 4]. The widespread adoption of smartphones and emergence of numerous apps marketed to MSM users has accelerated the popularity of online venues for MSM to connect with each other and in recent years, a very large proportion of MSM report meeting sexual partners online through dating websites or apps [5–7].

Numerous studies, as compiled in one meta-analysis, have explored HIV and sexually transmitted infection (STI) transmission risk among app-using MSM [8]. There is some evidence that MSM who use apps to meet partners are more likely to engage in condomless anal sex, have a higher number of sex partners and experience higher incidence of STIs, such as gonorrhea and chlamydia, as compared to those who meet partners in-person exclusively [9, 10]. Other studies are more equivocal and have found that meeting partners online is not predictive of sexual risk behaviors [11–14]. It is also unclear whether meeting partners online promotes high-risk behaviors or merely facilitates these behaviors among those who would be engaging in it regardless of their app use [15, 16]. Furthermore, previous studies indicate a correlation between app use and increased uptake of sexual health resources, including HIV testing and use of pre-exposure prophylaxis (PrEP) [17, 18]. App users are also more likely to be offered risk assessments for HIV and STI transmission during visits with their healthcare providers [19]. With a high volume of users, an efficient way to facilitate meeting new partners, expand sexual networks and connect users to prevention interventions and messaging, these apps have many features that can facilitate transmission as well as reduce it through providing structural features which facilitate communication among users regarding sexual health.

With the advent of new technologies for identifying and meeting partners, there are new opportunities for MSM to exchange information with partners by using profile options before meeting and/or engaging in sexual activity. Dating apps have increased the number of specific fields for users to share information, both as the apps observe that multiple users are populating open fields with specific sexual health information, and due to advocacy from Building Healthy Online Communities, a consortium of public health organizations [20–22]. Sexual health profile options vary from app to app, but usually include some or all of the following: disclosure of HIV status, usage of PrEP, having an undetectable viral load, willingness to use condoms, and date of last HIV and/or STI test. They provide a useful alternative for individuals who may be reticent or find it awkward to discuss these topics verbally with a partner. This can also inform and facilitate risk sorting among men who wish to make a conscious choice about which partners to approach with regards to their sexual practices. In contrast to serosorting, which requires individuals to know their HIV status, users can and do use risk sorting by seeking out partners based on their sexual practices. For example, through viewing others’ profiles, a user may or may not choose to have sex with someone based on their sexual health strategies and practices, as well as drug and alcohol usage. Since transmission through a sexual network in part depends on the degree to which users who take occasional risks have sex with someone who takes frequent risks [23], facilitating explicit communication can help reduce unintentional mixing. Profile options also increase communication without relying on ongoing public health human and economic resources to support costly behavioral interventions, which often have low uptake and may reach only a small percentage of app users [24].

As an example, diffusion of innovations theory [25] suggests that app users may be more likely to use PrEP if they see others including it on their profiles. A qualitative study that recruited Black MSM through geo-social apps found that dating apps were a common way for participants to learn about PrEP [26]. Another study found that 42% of HIV-negative men and 62% of HIV-positive men had partners they had met through a dating app who disclosed their PrEP usage [27], leading the researchers to draw the conclusion that mobile apps may aid with disclosure of both PrEP and undetectable viral load. While many may benefit from additional information and explicit sexual health communication, these fields are all optional. Giving users the choice of whether or not to disclose behaviors or sexual health risk strategies on apps is important to protect users who may be particularly vulnerable to stigma and persecution.

Building Healthy Online Communities (BHOC) is a consortium of public health leaders and gay dating website and app owners working together to support HIV and STI prevention online. BHOC focuses on structural changes to the apps (such as changing or adding features in the apps) and access to sexual health resources that can support dating app users’ sexual health. BHOC has developed ongoing relationships with app owners, and collected data from them to assess their willingness to implement these features. Additionally, BHOC has repeatedly sought input through focus groups and surveys to assess the willingness of app users to utilize them. Collecting data from owners and users is essential for the development of online sexual health promotion strategies.

A formative research survey in 2009 indicated strong levels of support among owners of online dating sites for integrating prevention options into their structures [27]. Additionally, we found that 78% of MSM users supported the integration of prevention options. These include allowing users to post their sexual health prevention strategies on profiles, facilitating online partner notification options, implementing testing reminders, and providing access to sexual health information and resources. We found that 80% of users supported having profile options that include HIV status disclosure [20]. More recently, in 2018, a University of Washington study confirmed continued user support for sexual health features, including access to home testing and reminders about sexual health testing [28]. In the present study, we use data from the American Men’s Internet Survey (AMIS), a large nationwide online survey of US MSM, to assess awareness and uptake of these features among MSM who use dating apps and analyze differences by demographic and behavioral characteristics.

## Methods

### Recruitment and Enrollment

AMIS conducts data collection annually and aims to collect 10,000 complete surveys from eligible MSM each year and AMIS recruitment methods have been previously reported [29]. Data for the present analysis were taken from the 2018 cycle, which ran from September to November 2018 [30]. Briefly, participants were recruited through convenience sampling from a variety of websites, including dating apps and general social networking websites such as Facebook and Instagram, using banner ads and email blasts to website members (hereafter referred to as “ads”). Participants who completed the survey in previous years were also asked to provide an email address to be invited to complete the present survey. Men who clicked on ads or emailed links were taken directly to the survey website hosted on a secure server administered by SurveyGizmo (Boulder, CO, USA). Participants were eligible to participate if they were age 15 years or older, reported male sex at birth and male gender identity, resided in the United States and provided a US ZIP code, and reported oral and/or anal sex with a male partner at least once in the past. Eligible participants were presented with an electronic informed consent form (or assent form, for those ages 15 to 17) and asked to affirm consent or assent by checking a box in the survey. No incentive was provided for completing the survey.

Several data cleaning steps were applied to the dataset of eligible responses, as described previously [30]. First, to deduplicate survey responses, demographic data for near-complete (> 70%) survey responses with non-unique internet protocol (IP) addresses were compared, and responses that showed a 100% match for all characteristics were considered to be duplicate responses. Only the observation with the highest survey completion was retained. Then the dataset was limited to those surveys that were deemed successful, i.e., observations with no missing values for the first question of at least two consecutive sections. Finally, the dataset was restricted to include participants who reported having oral or anal sex in the past 12 months and who provided a valid US ZIP code.

### Human Subjects Protection

The study was conducted in compliance with federal regulations governing protection of human subjects and was reviewed and approved by Emory University’s Institutional Review Board. No incentive was provided to the participants. Study data are protected under a federal certificate of confidentiality that prevents legal action to force data release.

### Measures

The primary outcomes in the analysis were awareness and utilization of profile features, which was asked of those participants who earlier in the survey responded that they had used dating websites or mobile phone apps to meet or socialize with other men in the past year. Awareness was assessed with the question, “Do you know that on many apps you can include your preferred sexual health strategy, like condoms, PrEP, or being on treatment for HIV?” Among those who answered “Yes,” utilization was assessed with the question, “Did you opt into using the preferred sexual health strategy feature?” Participants who opted into using profile options were also asked about which specific options they displayed on their profiles: condoms, PrEP (for HIV-negative or unknown status participants) and/or taking HIV meds/being undetectable (for participants living with HIV). Participants who indicated that they were not aware of profile features were asked, “Is the preferred sexual health strategy feature one you would like to use in the future?” While not all apps offer all of these features, 85.2% of participants used the largest app which offers most of these options. Since participants were also asked if they would use the features, those who use apps without those features were still included.

Participant demographic characteristics included age, race/ethnicity, self-reported HIV status, primary language and county of residence population density. Self-reported HIV status was determined from responses to questions about having ever had an HIV test, results of the most recent HIV test and having ever had a positive HIV test. Participants were categorized as self-reported HIV-positive and HIV-negative or unknown status. Participant county of residence was determined from self-reported ZIP code and assigned a population density level from the National Center for Health Statistics (NCHS) 2013 rural–urban classification scheme [31]. We further collapsed these categories into a four-level urbanicity variable: urban (central), suburban (fringe), medium/small metropolitan, and rural (micropolitan and noncore) [32, 33].

Behavioral characteristics were assessed for the 12 months preceding the survey date and included condomless anal intercourse (CAI), number of male oral and/or anal sex partners and partner type. Partner type was categorized as either only main partners (someone the participant felt committed to above anyone else), only casual partners (someone the participant didn’t feel committed to or didn’t know very well) or both main and casual partners. For HIV-negative or unknown status participants, we assess use of PrEP in the past 12 months. Current antiretroviral therapy (ART) use was assessed for participants who reported living with HIV.

### Analyses

Eligible and consented participants were included in analyses if they had an unduplicated IP address, completed the survey, ever had oral and/or sex with a man in the past 12 months and provided a valid US ZIP code. The analysis sample was further restricted to those who reported using dating websites or mobile phone apps to meet other men in the past 12 months and who answered the survey question about awareness of profile options.

Descriptive statistics of awareness and use of profile options are presented for the analysis sample, and by participant demographic and behavioral characteristics. Frequencies of each type of profile feature (condoms, PrEP and being on HIV medication) reported were calculated among those who reported awareness of profile options.

Modified Poisson models were used to calculate prevalence ratios (PR) and 95% confidence intervals (CI) for bivariate associations between awareness of profile options and demographic and behavioral characteristics, among the total analysis sample and by HIV status. Characteristics that were significantly associated with awareness were included in a multivariable model to calculate adjusted prevalence ratios (aPR) and 95% CI. Similar models were used to calculate PRs and aPRs for use of profile options. Statistical significance was determined at P < 0.05. All analyses were performed using SAS 9.4 (Cary, NC).

## Results

Of the 10,129 MSM who answered the AMIS-2018 survey, 6737 (66.5%) reported using dating websites or apps to meet other men in the past 12 months. Of those respondents who answered the questions about awareness of profile options, approximately three quarters (4993/6525, 76.5%) responded that they were aware of profile options to indicate preferred sexual health strategies (Table 1). In a multivariable model, age was significantly associated with awareness, with those in the 30–39 age group having the highest awareness as compared to other age groups, and those age 24 and under having the lowest awareness. Hispanic ethnicity, as compared to non-Hispanic white race, was associated with lower awareness [adjusted prevalence ratio (aPR) 0.95; 95% Confidence Interval (CI) 0.91, 0.99]. However, overall, the differences in prevalence of awareness between racial and ethnic groups were small (see Table 1). Other characteristics positively associated with awareness of profile options included positive HIV status (aPR 1.09; 95% CI 1.05, 1.13), recent condomless anal sex (aPR 1.09; 95% CI 1.05, 1.13), having two or more sex partners in the past year (aPR 1.09; 95% CI 1.05, 1.13), and having only casual partners (aPR 1.08; 95% CI 1.02, 1.14) or both main and casual partners (aPR 1.07; 95% CI 1.01, 1.13). In a separate model that included only participants who were HIV-negative or of unknown status (Table 2), PrEP use in the past year was associated with increased awareness of profile options (aPR 1.16; 95% CI 1.13, 1.20). Among participants living with HIV, there were no significant differences in prevalence of awareness between those who were currently taking HIV antiretroviral medication as compared to those who were not (334/374, 89.3% vs. 32/35, 91.4%, respectively; PR 0.98; 95% CI 0.88, 1.09).

**Table 1:** Correlates of awareness of dating application profile options among US MSM who used dating websites or apps to meet men in the past 12 months, American Men’s Internet Survey, 2018

	N	Aware of profile options n (%)	PR (95% CI)	aPR (95% CI)^b^
Total	6525	4993 (76.5)		
Age				
15–17	320	178 (55.6)	**0.68 (0.61, 0.75)**	**0.73 (0.66, 0.81)**
18–24	2431	1682 (69.2)	**0.84 (0.81, 0.87)**	**0.88 (0.85, 0.91)**
25–29	860	703 (81.7)	0.99 (0.96, 1.03)	1.02 (0.98, 1.06)
30–39	951	815 (85.7)	**1.04 (1.01, 1.08)**	**1.06 (1.03, 1.10)**
40 and older	1963	1615 (82.3)	Ref	Ref
Race/Ethnicity				
Black, non-Hispanic	325	245 (75.4)	0.96 (0.90, 1.03)	0.95 (0.89, 1.02)
Hispanic	1047	750 (71.6)	**0.92 (0.88, 0.95)**	**0.94 (0.91, 0.98)**
White, non-Hispanic	4561	3570 (78.3)	Ref	Ref
Other or multiple races	488	349 (71.5)	**0.91 (0.86, 0.97)**	0.95 (0.89, 1.00)
County population density				
Urban	2405	1896 (78.8)	Ref	Ref
Suburban	1367	989 (72.3)	**0.92 (0.88, 0.95)**	**0.94 (0.90, 0.98)**
Small/medium metro	2141	1643 (76.7)	0.97 (0.94, 1.00)	0.99 (0.96, 1.02)
Rural	608	463 (76.2)	0.97 (0.92, 1.01)	0.99 (0.94, 1.04)
Primary language				
English	6344	4864 (76.7)	Ref	
Spanish	101	72 (71.3)	0.93 (0.82, 1.05)	
Another language	50	33 (66.0)	0.86 (0.71, 1.05)	
HIV status				
Positive	414	370 (89.4)	**1.18 (1.14, 1.22)**	**1.09 (1.05, 1.13)**
Negative/Unknown	6111	4623 (75.7)	Ref	Ref
Condomless anal sex in past 12 months				
Yes	4661	3688 (79.1)	**1.13 (1.09, 1.17)**	**1.09 (1.05, 1.13)**
No	1864	1305 (70.0)	Ref	Ref
Sex partners in past 12 months				
One	911	580 (63.7)	Ref	Ref
Two or more	5482	4318 (78.8)	**1.24 (1.18, 1.30)**	**1.12 (1.06, 1.19)**
Partner type in past 12 months				
Main only	834	562 (67.4)	Ref	Ref
Casual only	2506	1913 (76.3)	**1.13 (1.08, 1.19)**	**1.08 (1.02, 1.14)**
Main and casual	2982	2374 (79.6)	**1.18 (1.12, 1.24)**	**1.07 (1.01, 1.13)**

Bolded items indicate those that are statistically significant

**Table 2:** Correlates of awareness of dating application profile options among US HIV-negative or unknown status MSM who used dating websites or apps to meet men in the past 12 months, American Men’s Internet Survey, 2018

	N	Aware of profile options n (%)	PR (95% CI)	aPR (95% CI)^b^
Total	6525	4993 (76.5)		
Age				
15–17	319	177 (55.5)	**0.68 (0.62, 0.76)**	**0.75 (0.67, 0.83)**
18–24	2405	1661 (69.1)	**0.85 (0.82, 0.88)**	**0.90 (0.87, 0.94)**
25–29	830	675 (81.3)	1.00 (0.96, 1.04)	1.03 (0.99, 1.07)
30–39	881	749 (85.0)	**1.05 (1.01, 1.09)**	**1.06 (1.02, 1.10)**
40 and older	1676	1361 (81.2)	Ref	Ref
Race/Ethnicity				
Black, non-Hispanic	277	205 (74.0)	0.96 (0.89, 1.03)	0.96 (0.90, 1.04)
Hispanic	996	704 (70.7)	**0.91 (0.87, 0.95)**	**0.94 (0.90, 0.98)**
White, non-Hispanic	4271	3308 (77.5)	Ref	Ref
Other or multiple races	469	332 (70.8)	**0.91 (0.86, 0.97)**	0.95 (0.90, 1.01)
County population density				
Urban	2212	1728 (78.1)	Ref	Ref
Suburban	1281	910 (71.0)	**0.91 (0.87, 0.95)**	**0.94 (0.90, 0.98)**
Small/medium metro	2034	1545 (76.0)	0.97 (0.94, 1.00)	1.00 (0.97, 1.03)
Rural	581	439 (75.6)	0.97 (0.92, 1.02)	1.00 (0.95, 1.06)
Primary language				
English	5938	4500 (75.8)	Ref	
Spanish	96	67 (69.8)	0.92 (0.81, 1.05)	
Another language	49	33 (67.3)	0.89 (0.73, 1.08)	
Condomless anal sex in past 12 months				
Yes	4309	3372 (78.3)	**1.13 (1.09, 1.17)**	**1.06 (1.03, 1.10)**
No	1802	1251 (69.4)	Ref	Ref
Sex partners in past 12 months				
One	884	555 (62.8)	Ref	Ref
Two or more	5100	3978 (78.0)	**1.24 (1.18, 1.31)**	**1.12 (1.05, 1.19)**
Partner type in past 12 months				
Main only	803	535 (66.6)	Ref	Ref
Casual only	2329	1754 (75.3)	**1.13 (1.07, 1.19)**	**1.07 (1.01, 1.14)**
Main and casual	2783	2197 (78.9)	**1.18 (1.12, 1.25)**	1.06 (0.99, 1.12)
PrEP use in past 12 months				
Yes	1068	976 (91.4)	**1.27 (1.24, 1.30)**	**1.16 (1.13, 1.20)**
No	4892	3527 (72.1)	Ref	Ref

Bolded items indicate those that are statistically significant

Just over half (869/1532, 56.7%) of participants who were not aware of profile options responded that they would like to use the preferred sexual health strategy features in the future. A substantial proportion of unaware participants (207/1532, 13.5%) indicated that they did not know if they would use these features.

The prevalence of using profile options among those who were aware of them was 61.1% (2866/4721) (Table 3). In a multivariable model, living in a small/medium metropolitan or rural county was negatively associated with using profile options (aPR 0.92; 95% CI 0.87, 0.97 and aPR 0.90, 95% CI 0.83, 0.99, respectively). Having two or more sex partners in the past year was positively associated with using profile options (aPR 1.17; 95% CI 1.06, 1.29). Among participants who were HIV-negative or of unknown status, PrEP use in the past year (Table 4) was associated with increased use of profile options (aPR 1.48; 95% CI 1.42, 1.56). Among participants living with HIV, there were no significant differences in use of profile options between those who were currently taking HIV antiretroviral medication as compared to those who were not (204/322, 63.4% vs. 16/30, 53.3%, respectively; PR 1.19; 95% CI 0.84, 1.68).

**Table 3:** Correlates of dating application profile options use among US MSM who used dating websites or apps to meet men in the past 12 months and were aware of profile options, American Men’s Internet Survey, 2018

	N	Use of profile options n (%)	PR (95% CI)	aPR (95% CI)
Total	4721	2886 (61.1)		
Age				
15–17	159	92 (57.9)	0.93 (0.81, 1.07)	
18–24	1580	930 (58.9)	0.95 (0.89, 1.00)	
25–29	676	400 (59.2)	0.95 (0.88, 1.02)	
30–39	775	510 (65.8)	1.06 (0.99, 1.13)	
40 and older	1531	954 (62.3)	Ref	
Race/Ethnicity				
Black, non-Hispanic	230	137 (59.6)	0.97 (0.87, 1.08)	
Hispanic	709	420 (59.2)	0.96 (0.90, 1.03)	
White, non-Hispanic	3393	2086 (61.5)	Ref	
Other or multiple races	323	199 (61.6)	1.00 (0.92, 1.10)	
County population density				
Urban	1798	1148 (63.8)	Ref	Ref
Suburban	936	583 (62.3)	0.98 (0.92, 1.04)	0.97 (0.92, 1.04)
Small/medium metro	1552	907 (58.4)	**0.92 (0.87, 0.97)**	**0.92 (0.87, 0.97)**
Rural	433	248 (57.3)	**0.90 (0.82, 0.98)**	**0.90 (0.83, 0.99)**
Primary language				
English	4600	2821 (61.3)	Ref	
Spanish	69	34 (49.3)	0.80 (0.63, 1.02)	
Another language	29	18 (62.1)	1.01 (0.76, 1.35)	
HIV Status				
Positive	356	221 (62.1)	1.02 (0.93, 1.11)	
Negative/Unknown	4365	2665 (61.1)	Ref	
Condomless anal sex in past 12 months				
Yes	3501	2145 (61.3)	1.01 (0.96, 1.06)	
No	1220	741 (60.7)	Ref	
Sex partners in past 12 months				
One	544	282 (51.8)	Ref	Ref
Two or more	4088	2550 (62.4)	**1.20 (1.11, 1.31)**	**1.17 (1.06, 1.29)**
Partner type in past 12 months				
Main only	528	292 (55.3)	Ref	Ref
Casual only	1794	1091 (60.8)	**1.10 (1.01, 1.20)**	1.03 (0.93, 1.13)
Main and casual	2267	1425 (62.9)	**1.14 (1.05, 1.23)**	1.04 (0.95, 1.14)

Bolded items indicate those that are statistically significant

**Table 4:** Correlates of dating application profile options use among US HIV-negative or unknown status MSM who used dating websites or apps to meet men in the past 12 months and were aware of profile options, American Men’s Internet Survey, 2018

	N	Use of profile options n (%)	PR (95% CI)	aPR (95% CI)
Total	4365	2665 (61.1)		
Age				
15–17	158	91 (57.6)	0.93 (0.81,1.07)	
18–24	1560	917 (58.8)	0.95 (0.89,1.01)	
25–29	648	388 (59.9)	0.97 (0.90,1.04)	
30–39	714	473 (66.2)	1.07 (1.00,1.14)	
40 and older	1285	796 (61.9)	Ref	
Race/Ethnicity				
Black, non-Hispanic	190	115 (60.5)	0.99 (0.88,1.11)	
Hispanic	664	395 (59.5)	0.97 (0.91,1.04)	
White, non-Hispanic	3142	1926 (61.3)	Ref	
Other or multiple races	307	188 (61.2)	1.00 (0.91,1.10)	
County population density				
Urban	1637	1048 (64.0)	Ref	Ref
Suburban	861	535 (62.1)	0.97 (0.91,1.03)	1.02 (0.96,1.09)
Small/medium metro	1455	846 (58.1)	**0.91 (0.86,0.96)**	0.97 (0.92,1.03)
Rural	411	236 (57.4)	**0.90 (0.82,0.98)**	0.98 (0.89,1.07)
Primary language				
English	4249	2601 (61.2)	Ref	
Spanish	65	33 (50.8)	0.83 (0.65,1.05)	
Another language	29	18 (62.1)	1.01 (0.76,1.35)	
Condomless anal sex in past 12 months				
Yes	3196	1958 (61.3)	1.01 (0.96,1.07)	
No	1169	707 (60.5)	Ref	
Sex partners in past 12 months				
One	519	267 (51.4)	Ref	Ref
Two or more	3762	2346 (62.4)	**1.21 (1.11,1.32)**	**1.13 (1.02,1.26)**
Partner type in past 12 months				
Main only	501	276 (55.1)	Ref	Ref
Casual only	1641	998 (60.8)	**1.10 (1.01,1.21)**	1.01 (0.92,1.11)
Main and casual	2098	1317 (62.8)	**1.14 (1.05,1.24)**	0.99 (0.90,1.09)
PrEP use in past 12 months				
Yes	951	776 (81.6)	**1.48 (1.42, 1.55)**	**1.46 (1.40,1.53)**
No	3316	1826 (55.1)	Ref	Ref

Bolded items indicate those that are statistically significant

Of all participants who included any sexual health strategy on their profile, approximately half (1430/2886, 49.5%) indicated that they used condoms. The vast majority (665/776, 85.7%) of participants who took PrEP in the past year and opted into any profile options included PrEP use on their profile. Two-thirds (136/204, 66.7%) of participants who took HIV antiretroviral therapy and opted into any profile options included HIV medications/undetectable viral load on their profile.

## Discussion

In a national, cross-sectional survey of internet-using MSM, we found a high degree of awareness and use of dating profile options to disclose preferred strategies for decreasing HIV and STI transmission risk. There was some demographic variability in knowledge of and uptake of these profile options, with users under the age of 25 reporting significantly lower awareness. As young MSM are a highly vulnerable population with respect to HIV and STI infection [34, 35] and also use dating apps to meet partners at higher rates than older MSM [8], increasing knowledge of these options may benefit young MSM by increasing sexual health communications between potential partners. Indeed, among those MSM who were aware of profile options, use of these options was not significantly different among younger users as compared to other age groups. We also found slightly lower awareness among Hispanic users. Although participant language was not found to be significantly associated with knowledge of these profile options, this may be due to the survey only being administered in English, and the number of users who indicated a primary language other than English was small. Those who lived in more rural areas were less likely to indicate use of profile options, possibly attributable to privacy concerns in areas with smaller populations of MSM. However, these differences were minor. Thus, these findings reveal high levels of acceptance among a large proportion of dating app users, and that many users may benefit from increased uptake of these dating profile options [27].

More substantial differences in uptake were found regarding HIV status and self-reported sexual behaviors. The greatest proportion of app users who were aware of the sexual health profile options were those who reported taking PrEP. This may be due to their interest in having sex without condoms, which may increase their desirability in the sexual marketplace among some gay men [36–38] or to communicate their health strategy to others. Usage was also highest among PrEP users, and of all specific profile options that we asked about, PrEP use was the feature that had the highest level of uptake. Awareness of profile options was significantly higher among participants who reported living with HIV, engaged in condomless anal sex, or had two or more partners and had casual sex partners. Usage was significantly higher only among those with multiple partners. This may be due to higher usage generally of apps among those who have multiple sex partners over the course of a year. Notably, no significant differences in awareness or use were found among participants living with HIV who were on ART, as compared to those who were not, but this may be due to the small number of HIV-positive participants who were not taking ART. There may be many motivations for disclosing HIV status; for example, in formative surveys gathered by BHOC, many HIV-positive users indicated wanting to identify other HIV-positive partners.

Another key finding is that over half of the participants who were not aware of profile options were interested in using them. This suggests that increased adoption of these features, especially among younger users, could be achieved by the apps increasing awareness of the options and their benefits through promotional efforts.

Dating apps are now used by the vast majority of MSM [7, 39, 40], and with their large number of users, they present an opportunity for intervention to reduce HIV and STI transmission at the population level. Much like gay bars and sex clubs have served as partners for behavioral and structural HIV education and prevention efforts since the earliest days of the epidemic [41, 42], dating apps for MSM can play a critical role in these efforts, especially in the context of a decline in physical venues in which MSM socialize [43].

With 67% of MSM reporting app use within the past year to meet a partner, nearly 2.6 million of the estimated 4 million MSM in the US could benefit from increased utilization of the sexual health options offered on the apps [33]. While our findings indicate significant uptake among MSM to an estimated 1.1 million users, there is still much room for improving uptake. The higher the proportion of app users who complete these fields, the easier it will be to normalize the sharing of sexual health information, and the easier it will be for app users to identify partners whose prevention preferences match their own.

It is essential to point out that these fields are all optional, and that completing them may not always be in the best interest of the user. HIV-related stigma remains prevalent within MSM communities [44]. Many people may be uncomfortable publicly posting personal information about their sexual preferences and health due to concerns over privacy and unintended disclosure to other parties, especially within communities with high levels of sexual identity stigma [45] and younger users who may not have disclosed their sexual identity to family or social circle [46]. Furthermore, the terms of service for apps generally prohibit users under the age of 18, which makes efforts to increase awareness of profile options among minors who access the apps challenging. MSM may also have perceived and real safety concerns about encountering judgement or harassment from other users on the app, and this may lead to their choice not to use some of these features [47]. Apps should provide information in prominent places within the app that allows users to make informed choices regarding posting their sexual health information. Having these fields helps to normalize information sharing, and still allows users to opt out of using them.

Users may not be truthful about their information on a profile or keep their information up-to-date. While there is no perfect way to ensure honesty, app users should always be encouraged to treat the information received through the profile as the beginning of further conversation, and ideally continue to exchange more information via text or in-person before meeting up and/or engaging in sexual activity. Since many users may change their sexual health strategies over time, apps should support the posting and updating of accurate information by encouraging users to get tested and to review, and where necessary update, their information every 3 months.

We note several limitations to the present study. First, these data were collected through an online convenience sampling approach and may not be generalizable to all MSM in the US or all MSM online. Second, in some cases our terminology may not have been clear to study participants. For example, the term “prevention strategies” may not have been a term that all participants know. Third, while participant language was not found to be significantly associated with knowledge of profile options, this may be due to the fact that the survey was only administered in English. Fourth, data from this survey were collected in 2018. While three years is a substantial amount of time in the context of rapidly changing dating app technology, we have noted few structural changes in sexual health profile features available in apps since that time. Finally, not all apps have the same profile options available to users. While most of the apps include sexual health options as part of users’ profiles, apps differ in which options are included, and the wording of each option.

## Conclusions

BHOC has used data from users to prioritize and promote strategies on dating apps that are sustainable and reach high numbers of users, and require no or little ongoing investment by public health programs in order to create environments for MSM that support health-promoting behaviors [48]. These include new sexual health profile fields, sexual health testing reminders, links to partner notification services, testing, and other sexual health resources. BHOC hopes to increase use of sexual health profile fields and access to sexual health information by promoting usage of profile fields and testing reminders among those apps which have already implemented these features, and partnering with more dating apps who have not yet implemented them. Additionally, BHOC is encouraging apps to provide links to health information in prominent locations on apps, so we can increase usage. This research provides guidance for understanding the factors associated with uptake that can be used to guide both public health communication and ongoing conversations with apps about their support of sexual health.

As we work to implement these changes, we recognize additional research using qualitative methods to better understand user concerns about sexual health fields will enhance our interpretation of the data. We also encourage future research to include epidemiological modeling to determine the percentage of sexual health profile options that need to be completed in order to have the maximum population-level impact on transmission, by facilitating users making informed choices regarding sexual health practices with new partners.
